# Temporal and spatial patterns of mitochondrial haplotype and species distributions in Siberian larches inferred from ancient environmental DNA and modeling

**DOI:** 10.1038/s41598-018-35550-w

**Published:** 2018-11-29

**Authors:** Laura S. Epp, Stefan Kruse, Nadja J. Kath, Kathleen R. Stoof-Leichsenring, Ralph Tiedemann, Luidmila A. Pestryakova, Ulrike Herzschuh

**Affiliations:** 1 Alfred Wegener Institute Helmholtz Centre for Polar and Marine Research, Periglacial Research Section, Telegrafenberg A43, 14473 Potsdam, Germany; 2 Institute for Biochemistry and Biology, University of Potsdam, Karl-Liebknecht-Str 24-25, 14476 Potsdam, Germany; 3 Department for Geography and Biology, North-Eastern Federal University of Yakutsk, Belinskogo 58, 67700 Yakutsk, Russia; 4 Institute of Earth and Environmental Science, University of Potsdam, Karl-Liebknecht-Str 24-25, 14476 Potsdam, Germany

## Abstract

Changes in species’ distributions are classically projected based on their climate envelopes. For Siberian forests, which have a tremendous significance for vegetation-climate feedbacks, this implies future shifts of each of the forest-forming larch (*Larix*) species to the north-east. However, in addition to abiotic factors, reliable projections must assess the role of historical biogeography and biotic interactions. Here, we use sedimentary ancient DNA and individual-based modelling to investigate the distribution of larch species and mitochondrial haplotypes through space and time across the treeline ecotone on the southern Taymyr peninsula, which at the same time presents a boundary area of two larch species. We find spatial and temporal patterns, which suggest that forest density is the most influential driver determining the precise distribution of species and mitochondrial haplotypes. This suggests a strong influence of competition on the species’ range shifts. These findings imply possible climate change outcomes that are directly opposed to projections based purely on climate envelopes. Investigations of such fine-scale processes of biodiversity change through time are possible using paleoenvironmental DNA, which is available much more readily than visible fossils and can provide information at a level of resolution that is not reached in classical palaeoecology.

## Introduction

Climate warming is expected to cause major shifts in boreal ecosystems^1^, which are currently a huge carbon repository^2,3^ and are experiencing some of the greatest climatic changes worldwide^4^. These shifts, in turn, are projected to cause large-scale vegetation-climate feedbacks, in particular as albedo decreases with increasing forest cover^5^, which has been proposed as a major cause of the strong Arctic warming that occurred during the mid-Pliocene^6^. The Siberian boreal larch forests encompass over 260 million ha^7^, and both the extent and distribution of forested area^8^, as well as the distribution of the major forest-forming larch (*Larix*) species are projected to change^9^. The three recognized larch species, *Larix sibirica* Ledeb., *Larix gmelinii* (Rupr.) Kuzen. and *Larix cajanderi* Mayr., differ in a number of features, both in morphology and ecology^7^. Understanding and reliably forecasting shifts in their distributions is therefore highly relevant to the projection of future vegetation-climate feedbacks^5^.

**Figure 1:**
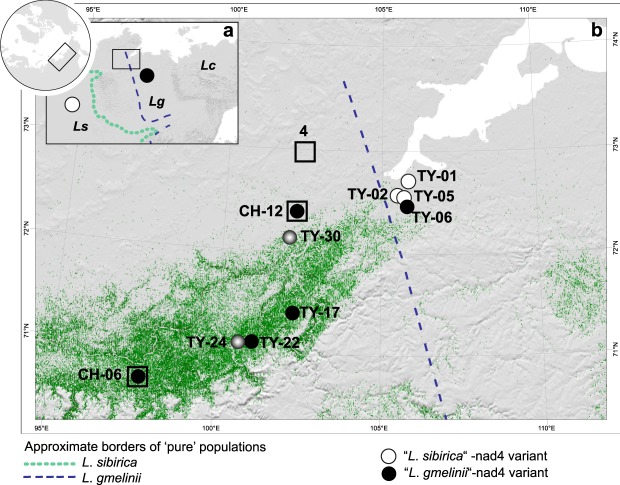
Study area and sites analysed in this study, and current distribution of mt DNA variants. **(a)** Broad-scale distribution of the different larch species *Larix sibirica* (*Ls*), *Larix gmelinii* (*Lg*) and *Larix cajanderi* (*Lc*), according to^7,11^ along with the previously published large scale distribution of the mt DNA variants (white and black dots). The study area is marked by a rectangle. **(b)** Sites analysed in this study, along with results from lake surface sediment DNA analyses for the *Larix-*specific mitochondrial nad4 amplicon. At sites CH12 and CH06, we retrieved two sediment cores, for which downcore data is presented in Fig. 2. From all other sites, the top centimetre of lake sediment was analysed. The shading of the dots indicates, which haplotype was indentified from the top sediment and matches the core samples in Fig. 2 (white = *L. sibirica*; black = *L. gmelinii*; gradient = both). The green shading indicates the density of trees taller than 5 m in height, as published by^64^. The dashed line indicates the previously published boundaries between *L*. *sibirica* and *L*. *gmelinii*^11^, with pure populations of *L*. *gmelinii* postulated to occur to the east of the dashed line. Sites marked with an open square were used in the modelling approach through time (Fig. 4), with site 4 near the top of the figure also used in the models of current populations (Fig. 3).

The Siberian larch species are distributed from west to east in large, fairly discrete ranges (Fig. 1), with hybridisation occurring in the boundary regions^7,10^. It is hypothesized that a natural invasion of *L*. *gmelinii* into the area of *L*. *sibirica* has occurred during the Holocene^11^, and a better understanding of this process can elucidate the dynamics of possible future range shifts of Siberian larches. Phylogeographic structure among and within the larch species is apparent using both chloroplast (cp) and mitochondrial (mt) genetic markers^10,11^. While precise delineation of species’ borders is not possible with either organellar DNA, published datasets^10,11^ include mt DNA loci that reflect the respective ranges of *L*. *sibirica* and *L*. *gmelinii* quite well on large spatial scales. Contrary to expectations^12–14^, patterns of cp DNA, which in Pinaceae is paternally transmitted through pollen and should exhibit high gene flow^15^, are not more effective in delimiting species’ ranges than the seed-dispersed mt DNA^10^.

As vegetation is classically perceived as being determined largely by climate^16^, existing projections have been based purely on climate envelopes. These suggest general range shifts of the different species to the north-east along with rising temperatures^9^. However, these projections do not account for the dynamics of change, which will also depend on the current distribution and migration potential (biogeographical factors) of the species, as well as biotic interactions^17^, such as competition and hybridisation of closely related species. In addition, natural disturbances, in particular wildfires and insect outbreaks are important determinants of boreal forest dynamics^18,19^. Each of the respective factors will have caused differences in past as well as present distributions, providing indications of their relative importance.

When species ranges are driven by climate, species will occupy all of their potential range, irrespective of the presence of other species. Species abundances will vary along climatic gradients, with boundary areas showing a gradual shift of species’ occurrence. Temporal climatic changes will directly induce range shifts. When the main driver is biogeography, current disributions will be closely linked to past distributions, and long-term records will track the arrival of a species into an area, after which it should persist. If the system is driven by competition, the current distribution will not necessarily trace the climatic optima of the species. Instead, the distribution of the species will be linked to the density of tree stands, as this determines the strength of competitive interactions. Through time, we can expect to see species turnover, similar to patterns in climate-driven systems, but the turnover will not be linked directly to climatic changes. If hybridisation is prevalent in boundary areas, range shifts will cause patterns of genomic introgression in the participating populations. This occurs preferentially from the local population to the invading population and for markers with low gene flow^20^. For Pinaceae such as *Larix* it is assumed that introgressed populations in boundary regions should carry the seed-transmitted mt DNA of the originally present species along with the pollen-transmitted cp DNA^15^ of the invading species. Through time, this should cause a change in the composition of cp DNA variants, whereas the mt DNA should remain unchanged. If the species’ distribution is strongly affected by local natural disturbances, the boundary area should not exhibit a clear pattern, but a mosaic of species’ presence.

Importantly, the patterns will not be fully visible in investigations focussing only on current distributions. Instead, potential turnover patterns can be revealed by conducting spatio-temporal analyses in boundary regions that have undergone historical changes and which ideally span gradients of both climate and population density. This is the case in the northern boundary zone of *L*. *sibirica* and *L*. *gmelinii*, located on the southern Taymyr peninsula in eastern Siberia. This region hosts the northernmost forests in the world and constitutes a wide treeline ecotone in which the tree density changes gradually from the more southerly light, northern taiga forests to the northern single-tree tundra over a distance of up to 200 km^21^. Historical fluctuations in forest extent and density are documented by macrofossils^22,23^ and pollen^24^, and future northward forest expansion is expected^25^, but information below the level of the genus *Larix* is lacking to date. Such information can be obtained from long time series, for example, from lake sediment cores^26^, and from proxies with sufficiently high taxonomic resolution, such as, potentially, environmental DNA stored in the sediments^27^. It cannot be accomplished using traditional pollen analysis, as this only resolves *Larix* to genus level, but lake sediments contain DNA from plants and other organisms^28–30^, and accurately reflect general vegetation change through time^31^ and in space^32,33^.

An independent strategy to identify driving factors for the dynamics of species is to perform simulations with process-based numerical models that use species-specific parameters for relevant processes, such as growth, seed production and dispersal, establishment, and mortality. For larch populations, such simulations can be carried out using the individual-based spatially-explicit model ‘*Larix* Vegetation Simulator’ LAVESI^25^. This model was initially parameterised only for *L. gmelinii*, but it can be extended to accommodate the presence of a second larch species.

Here, we take two independent approaches to investigate the dynamics of current and past ranges of *L. gmelinii* and *L*. *sibirica*:

1. We generated empirical data on the occurrence of mitochondrial single nucleotide polymorphism variants in space and through time on the southern Taymyr peninsula (Fig. 1). For this marker, the distribution of the two variants on a broad spatial scale coincides very well with the distribution of *L*. *gmelinii* and *L*. *sibirica*^10,11^, and thus we use the terms “*L*. *gmelinii*”-variant and “*L*. *sibirica*”-variant. We analysed a series of lake surface sediments across the treeline ecotone (Fig. 1) and two lake sediment cores covering most of the Holocene from a northern (CH12) and a southern site (CH06). We compared inferred vegetation development based on sedimentary DNA metabarcoding^34^ with that from pollen analyses of the sediment cores. These analyses provided data on larch stand density through time.
2. We extended the individual-based spatially-explicit model LAVESI^25^ to include both *L*. *gmelinii* and *L*. *sibirica*. With this extended model, we performed simulations in space under current climatic conditions for a range of sites in north-central Siberia. We also ran experiments representing the last 6000 years in relation to climate series simulated for three different sites on the southern Taymyr peninsula.

## Results

**Figure 2:**
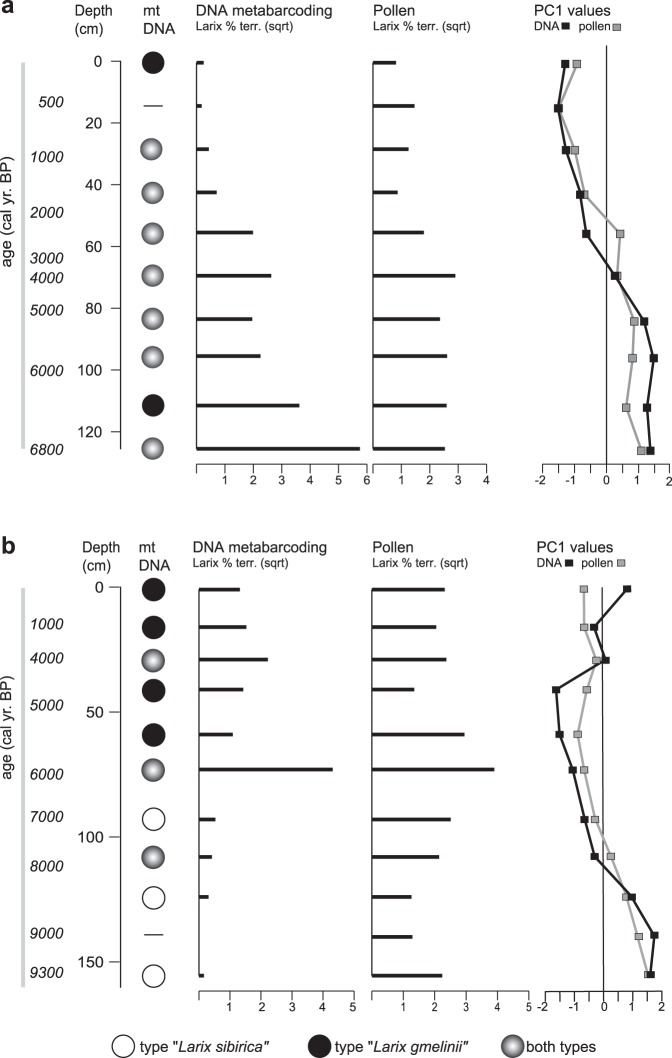
Stratigraphic plots of the two analysed cores CH12 **(a)** and CH06 **(b)**. The plots show the distribution of dominant *Larix* mitochondrial haplotypes, overall percentages of *Larix* in the DNA metabarcoding and pollen data (displayed as square root of raw counts), and first principal component values retrieved from principal component analyses (PCA) on the respective complete vegetation data. (Full stratigraphic plots and full PCA plots in Supplementary Figs S6–S9.)

The general vegetation history of the two analysed cores is similarly captured by plant DNA metabarcoding on the sedimentary DNA and by pollen analyses. Both approaches have similar trends in the first component of a principal component analysis (PCA) performed on the respective datasets (Fig. 2a,b; full PCAs in Supplement), which lends support to the general validity of the results obtained with sedimentary DNA.

The mitochondrial haplotypes retrieved from the series of lake surface sediments have a distinct distribution in space: the three most north-easterly lakes yielded exclusively the “*L*. *sibirica*”-variant, whereas the other lakes were either dominated by the “*L*. *gmelinii*”-variant (three lakes) or showed a mixed signal (two lakes, Fig. 1, see Methods and Supplement for details on our definition of dominance of the variants). This picture contrasts with previously published studies, which show that on a larger geographical scale, the “*L*. *sibirica*”-variant is found further to the west, whereas the “*L*. *gmelinii*”-variant is present to the east of the Taymyr peninsula^10,11^ (Fig. 1).

There is a notable presence of the “*L*. *sibirica*”-variant in a large part of the core from the northern site CH12 (Fig. 2a). In this core, which covers a timespan back to about 7000 cal years BP^24^, samples up to about 1000 cal years BP have both variants (Fig. 2a). Only the topmost sample and one sample from about 6300 cal years BP show a clear dominance of the “*L. gmelinii*”-variant. As each centimetre of sediment throughout the cores contains material from multiple decades (CH12 median = 39 years, CH06 median = 41 years), the co-occurence of variants can result either from true co-occurrence within the catchment of the lake, or from local population extinctions and re-establishments. The overall vegetation development revealed by DNA metabarcoding and pollen^24^ throughout the time period is characterised by a strong decline in forest cover, and current vegetation can be characterised as shrub tundra^24^ (Supplementary Figs S2 and S3).

In comparison, the core from the southern site CH06, which extends back to about 9000 cal years BP, is mostly dominated by the “*L*. *gmelinii*”-variant from about 5400 cal years BP onward. Samples older than about 7000 cal years BP are dominated by the “*L*. *sibirica*”-variant (Fig. 2b). The two lowest samples containing larch DNA, below an age of about 8500 cal years BP yielded exclusively sequences of the “*L. sibirica*”-variant. The shift in dominance from the “*L. sibirica*”-variant to the “*L*. *gmelinii*”-variant occurs just above a sample documenting a peak in larch presence in both DNA metabarcoding and pollen and with an age of about 6100 cal years BP. The overall Holocene vegetation development, as depicted by the DNA metabarcoding and the pollen data, is characterised by an increase in forest cover from 9000 cal years BP to 6100 cal years BP, followed by a drop in *Larix* densities, although forest persists up to the present day in the catchment of the sampled lakes (Supplementary Figs S4 and S5).

**Figure 3:**
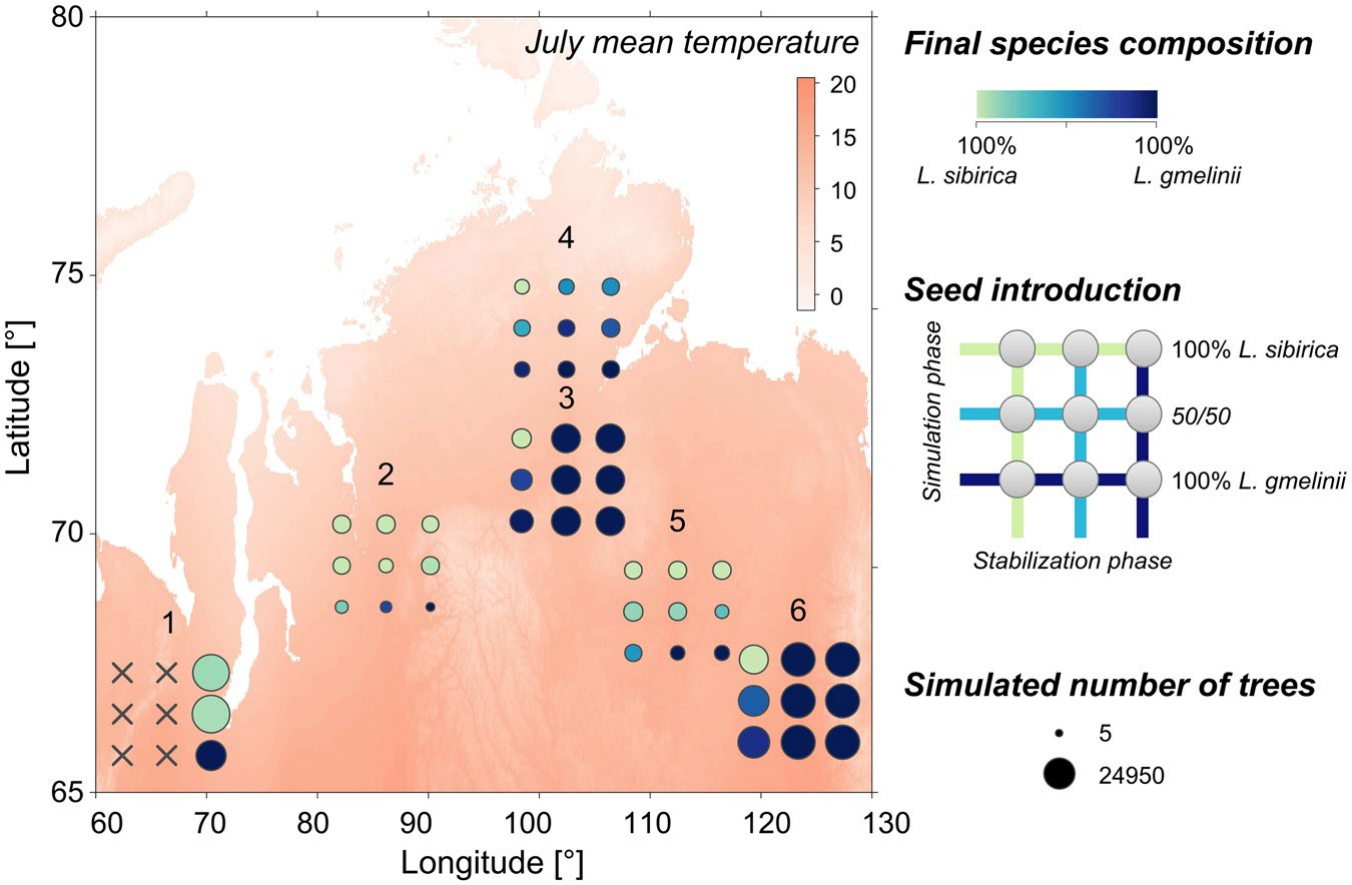
Spatial distribution of larch species in simulations with the model LAVESI. The 3 × 3 matrices show the different simulated recent species composition of *L. sibirica* and *L. gmelinii* on a colour gradient from light green to dark blue, with dot sizes equivalent to stand densities in number of trees per hectare, for differing seed introduction in the simulation phases. The westernmost site (1) could not be fully analysed because some simulation runs aborted due to impedingly large modelled population sizes

In our second line of investigation, we ran experiments with the process- and individual-based model LAVESI^25^ that simulated the current *Larix* populations in space in relation to simulated climate series generated from recent weather data (Fig. 3). We targeted sites with different current temperatures and inserted seeds of each of the two species, or of both species, in the stabilisation phase and the simulation phase (see legend in Fig. 3). Overall, results from simulations, in which both species were inserted matched their recognised distribution well^7^. When the species were introduced singly, each species was simulated to grow at all sites, and mostly to reasonable population sizes. A notable exception to this overall pattern was a population on the south-east of the Taymyr peninsula (site 5, Fig. 3), which in the model simulations was mostly dominated by *L*. *sibirica*, even though the site is within the published distribution range of *L*. *gmelinii*^7^.

In contrast to the other sites, the site at the northern edge of the study area (site 4, Fig. 3, and see Fig. 1) displayed a mixed population under a number of scenarios. Although climate at this northernmost site is harsh, and intuitively this implies more suitable conditions for *L*. *gmelinii*, *L*. *gmelinii* only dominated the simulated populations when it was inserted as the sole species in the simulation phase. The population sizes at this northern site remained small in all simulations.

**Figure 4:**
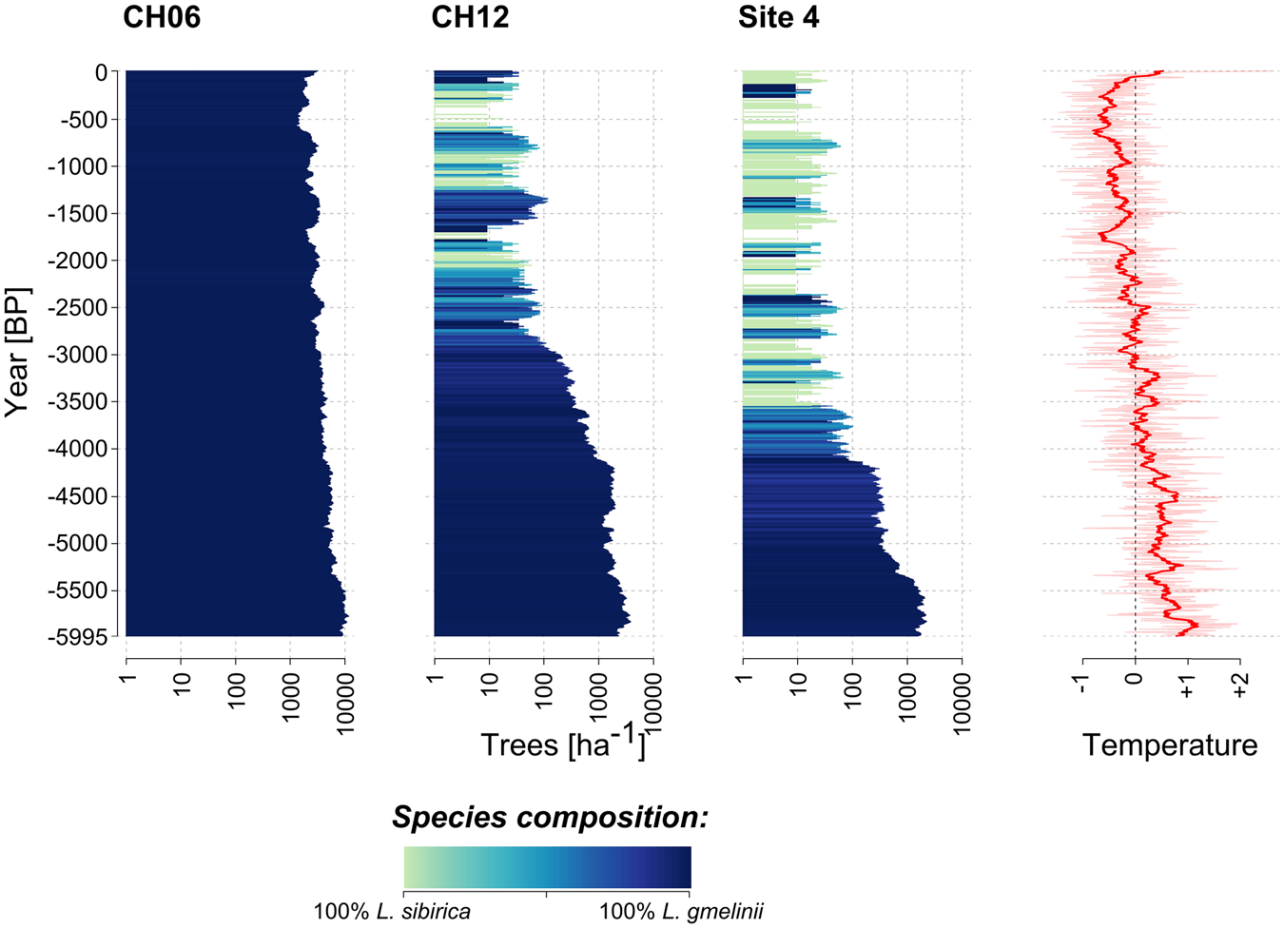
Temporal occurrence of the two larch species from simulations with the model LAVESI. Simulations were started by introducing seeds of both species into the simulated area 5996 years ago. The temperature series is based on simulation data of an ESM-run 6000 k^35^ and climate from the appropriate grid cell, standardized to the average temperature between 1960 and 1990. The mixed-seed introduction was continued during the simulation phase to allow new introductions after die-out events. At all three sites the simulated plots were first dominated by* L. gmelinii* and tree densities track the general decrease of temperature during the simulation phase, reaching their lowest point roughly 200 years ago. *L. sibirica* only emerged when densities dropped below ~100 trees ha^−1^ and as a result mixed stands were only simulated at the two northernmost sites.

A dependence of species occurrence on population size was also revealed in the temporal simulations of population dynamics. These covered the last 6000 years and were based on climate data simulated from an ESM-run 6000 k^35^ adjusted to the climate of our study area (see Methods). We ran simulations inserting both species for three sites in our study area: the two sites CH06 and CH12, from which we also analysed the lake sediment cores, and the northernmost site (4), for which simulations were run for current conditions. Site 4 is located well to the north of the current treeline, in an area where a harsh climate prevails and where single trees grow only sparsely in isolated groups on the tundra. In these simulations, the more southerly site CH06 was dominated by *L*. *gmelinii* throughout the entire period, while at the other two sites, the two larch species alternated or occured together for the final 4000 years (site 4) or 3000 years (site CH12), respectively (Fig. 4). At these sites a change from the dominance of *L*. *gmelinii* to both species being present occurred when the simulated population size dropped below about 100 trees/ha as a result of cooling climate.

## Discussion

The distribution of mt DNA variants in space and through time shows a clear link to forest density: (1) occurrence of the “*L*. *sibirica*”-variant is more likely at more northern sites, in which forest density is low, and (2) in the more southerly core, the originally occurring “*L*. *sibirica*”-variant is replaced by the “*L*. *gmelinii*”-variant after forest density increases. An equivalent dependence of species occurrence on population size is obtained through the simulations inserting the two different species. Our recovery of a spatial pattern implies that the species ranges are likely not driven by stochastic natural disturbances. The results also suggest that the distribution and dynamics of range shifts of Siberian larches is not linked directly to climatic factors, as assumed by simple species distribution or climate envelope models^36^.

Climate does play a role in the historical vegetation development, as it, to a large part, determines changes in stand density. While other natural disturbances, such as insect outbreaks and wildfires^37,38^, are important in causing population declines, and thus stand density fluctuations, warming temperatures cause increased productivity and growth rates of larches on Siberian permafrost^39,40^. For site CH12, a continuous vegetation turnover from open forest to single-tree tundra between 3000 and 2000 cal years BP has previously been documented and attributed to a summer temperature decline of about 2 °C over the last 7100 years^24^, which is in agreement with general temperature reconstructions for the Arctic^41^. This vegetation turnover is also revealed by the DNA metabarcoding data (Fig. 2a, Supplementary Fig. S2), but no concurrent change in the dominance of larch haplotypes is apparent. The core from site CH06 reaches back further in time and the lower part of the core depicts a *Larix* population increase in accordance with rising early Holocene temperatures^42^. The larch haplotype turnover between 6000 and 5000 cal years BP, however, seems to be more strongly linked to a large *Larix* population size than to climatic conditions.

The initial dominance of the “*L. sibirica*”-variant in the DNA data of core CH06, especially its exclusive presence in the lowest samples, is putatively an effect of biogeography. Previously published analyses of current populations have suggested the existence of a northern glacial refugium for *L*. *sibirica*^11^, and our mitochondrial haplotype data from CH06 lend direct support to the notion that our study area was populated by *L*. *sibirica* at the beginning of the Holocene. While our records do not reach beyond the Holocene, and we therefore cannot reconstruct the glacial distribution, the early postglacial retrieval of only the “*L*. *sibirica*”-variant is likely due to glacial populations persisting in close proximity.

This early postglacial haplotype distribution was not, however, maintained throughout the Holocene: instead we see a displacement of the “*L*. *sibirica*”-variant by the “*L*. *gmelinii*”-variant in core CH06 after larch populations had reached a maximum at about 6100 cal years BP. A natural historical invasion by *L*. *gmelinii* to the expense of *L*. *sibirica* in the area is a long-standing hypothesis^11,43^, resulting in a hybrid population^44^. According to theoretical expectations of assymetric introgression^13,20^, these populations should exhibit mt DNA of *L*. *sibirica* and cp DNA of *L*. *gmelinii*, and this has indeed been reported for extant trees in a previous study^11^, but with a relatively coarse spatial resolution. Contrary to these expectations for a scenario of introgression, our data do not show a long-term persistence of the “*L*. *sibirica*” mt DNA variant in either of the sediment cores. While we did not find any clear pattern of cp DNA variant changes, and thus cannot fully reconstruct the history of stand change, the complete displacement of mt DNA variants in the more southern core CH06 rather suggests a historical replacement of seeds, which carry the mitochondrial DNA.

Importantly, the spatial and temporal distribution of mt DNA variants (Figs 1 and 2) is linked to stand density, and this pattern is also recovered in simulations of populations of the two species (Figs 3 and 4). Such a density dependent distribution suggests that competition and competitive exclusion^45^ is the factor most strongly determining the dynamics of range shifts of *L*. *sibirica* and *L*. *gmelinii* in their boundary area at the treeline. These conclusions are reached independently by the different approaches we used, and for both current and past populations. The overall validity of the sedimentary DNA record is furthermore supported by the high degree of congruence of the metabarcoding data with the results from pollen analyses (Fig. 2). The study therefore demonstrates the utility of sedimentary DNA to gather empirical data on historical shifts in species and population ranges, which can be used to validate the accuracy of models and their projections^46^.

Our findings highlight that a reliance on climate as the driving factor for species’ distributions is not always adequate. For larches, previously published simulations suggest range shifts of the species to the north-east^9^, but our data suggest possible different outcomes. The historical vegetation development and current field assessments in the study area^21^ imply that the main effect of warming climate is an overall densification of the forests. This in turn should lead to an intensification of competition, which, under climate conditions that are not too dissimilar from the present, will favour an expansion of *L*. *gmelinii* in the area – an outcome directly opposed to that reached when considering only the climatic optima of the species^9^.

This can potentially have important implications for future vegetation-climate feedbacks. For example, the growth rates of *L*. *gmelinii* are substantially lower than those of *L*. *sibirica* (Supplementary Table S1)^47,48^, and it will presumably take longer for a stand of *L*. *gmelinii* seedlings to develop into a mature forest. Such slower biomass production would have negative effects for the attenuation of climate warming through carbon sequestration. At the same time, slower forest growth would limit decreases in albedo, which is considered the larger threat for positive climate forcing through vegetation^5^. Furthermore, the dominant plant species will potentially influence the depth of the active layer and and thus the amount of carbon that is released^49^. In contrast to *L*. *sibirica*, which is sensitive to permafrost, *L*. *gmelinii* has extremely shallow roots and its distribution is linked to the occurence of permafrost^7^. While this is generally attributed either to the exceptional permafrost tolerance of *L*. *gmelinii*, or even to a dependance on permafrost^40^, the larches also stabilise the permafrost itself^50^. This implies that a future expansion of *L*. *gmelinii* could in effect prevent permafrost thaw in the area and thus cause less carbon to be released from the soil.

These possible implications demonstrate the importance of incorporating relevant processes and drivers into future projections, rather than relying purely on current distributions. In the case of Siberian larch forests, our analysis of mitochondrial DNA isolated from lake sediment cores in combination with process-oriented modelling shows that reliable projections must account for species-specific reactions and dynamics of range shifts. Ancient environmental DNA is an archive of biodiversity changes that can uncover dynamics at a resolution that is not reached by any other proxy. It is now routinely retrieved from palaeoenvironmental archives from mid to high latitudes with relative ease^30,51,52^ and can thus play a prominent role in validating projections of climate change outcomes. The increasing availability of genomic data will potentially enable the identification of informative cp DNA markers^53^, which can be used to further understand the processes of introgression and displacement in the boundary areas.

## Methods

### Empirical data

#### Sampling

Historical data on historical vegetation and *Larix* variants was obtained from two lake sediment cores from the southern Taymyr peninsula collected in July 2011 from the following sites: lake CH12 (72.399°N, 102.289°E), located in the northern part of the treeline ecotone, currently surrounded by a vegetation of single-tree tundra, and lake CH06 (70.667° N, 97.716°E), located in the southern part of the treeline ecotone in the light northern taiga. The sediment cores were transported back to the Alfred Wegener Institute in Potsdam and stored at 7 °C until sampled for pollen, DNA, and radiocarbon dating. Surface sediment samples were taken in the field from the first centimetre of short sediment cores as described in^32^. Details on sampling for DNA and on sampling locations are compiled in the Supplementary Information.

#### Sediment core chronology

Age models for the two sediment cores were developed from radiocarbon dates of the described cores, corroborated by sedimentation rates of the last two centuries inferred from ^210^Pb/^137^Cs dating of parallel short cores. Details of the chronology of core CH12 have been described previously^24,54^. The chronology of core CH06 is also based on radiocarbon dates, and details are given in the Supplementary Information.

#### Molecular genetic analyses

DNA was extracted from sediment samples and amplified using (1) an assay targeting all vascular plants^55^, and (2) primers specific to *Larix*, targeting a short amplicon of 61 base pairs without primers around the variable position 1433 of the mitochondrial *nad4/3-4* amplicon. Sequence data were analysed using programs from the OBITools package (http://metabarcoding.org/obitools). Details of the procedures are given in the Supplementary Information, along with detailed results,

#### Pollen analysis

Samples for pollen analysis of core CH06 were taken at the same or neighbouring depths of the samples for DNA analysis, and prepared using standard procedures^56^. Pollen grains were analysed using a Zeiss Axioskop 40 light microscope at 400×magnification and identified based on published pollen atlases^57–59^ and the pollen reference collection of the AWI in Potsdam. For most samples, at least 300 terrestrial pollen (arboreal and non-arboreal) grains were counted and included in the pollen sum. In the two lowest samples, which had extremely low pollen densities, pollen grains were only counted to a number of 279 and 127 respectively. Pollen analysis of the core CH12 was performed by^24^, and we used the data for the equivalent depths of the DNA samples.

#### Statistical analyses

To investigate the major structure in the individual datasets, we performed Principal Component Analysis (PCA) for the pollen and the DNA sequence data from each of the cores, i.e. a total of four multivariate datasets. As we intended to extract the main historical developments of the vegetation comparatively for pollen and DNA metabarcoding, we performed the analyses on taxa transformed to genus level and considered only dominant taxa that were present at a minimum of 0.5% in at least two samples. To limit the influence of outliers, all DNA and pollen percentages were square-root transformed prior to computational analyses. Analyses were done in R v. 3.3.1^60^, using packages “vegan”^61^ and “rioja”^62^. The full PCA biplots are presented in the Supplementary Information, while the scores of the PC1 for DNA metabarcoding and pollen are plotted stratigraphically in Fig. 2. Full stratigraphical plots of the most important taxa in each of the datasets are presented in the Supplementary Information.

#### Modelling

To analyse competitive interactions between *L*. *gmelinii* and *L*. *sibirica*, we modified the individual-based spatially-explicit model LAVESI^25^, originally set up only for *L*. *gmelinii*, to include *L*. *sibirica*. The modifications of the model are detailed in the Supplementary Information. Briefly, this model, programmed in C++, simulates the dynamics of larch stands and traces individual trees and seeds in yearly time steps. Within each simulated yearly cycle, after initialisation and setting the relevant parameters and environmental variables, sub-models for growth, seed production, seed dispersal, establishment, and mortality/ageing are consecutively invoked. In the modified version used here, seeds and trees are differentiated into two species. Depending on the species, the trees carry a different set of characters, compiled from literature sources, which influence sub-models for growth, seed dispersal, establishment, mortality, and ageing. Simulations in LAVESI are run for a defined climate time-series (simulation phase), following a stabilisation period of 1000 years, in which the weather data is randomised (stabilisation phase). Further details of the modified model and model validation are described in the Supplement. With the modified LAVESI we set up two types of experimental runs: (1) current larch populations at different localities in Siberia were simulated using recent weather data from the gridded climate dataset CRU TS 3.23^63^, relying on the years 1934–2013 and (2) historical larch populations around our study area were simulated for three sites, with the experiments running for 5996 years until the year 2013. The simulations were forced using a monthly temperature mean and precipitation sum series based on simulation data of an ESM-run 6000 k^35^ and climate from the grid cell in which the weather station of the town Khatanga is situated (71.75°N, 102.25°E) in the following way: to conserve the real climate variability for temperature and precipitation data (i) 19-yr-long windows were extracted from real climate for the years 1934–2013, (ii) these windows were randomly bound together to create a series of the same length of the simulated series, i.e. running from −3982–1933, excluding the overlap for which real climate data is available (1934–2013), and finally (iii) adjusted by calculating the differences in their total mean values of each window in relation to its corresponding window in the simulated dataset. From this master series, extended by the real climate series over the years 1934–2013, we derived three series by adjusting the full series with the difference of the series mean at CRU TS grid cell at the location of interest (CH06 = 70.75°N, 97.75°E; CH12 = 72.25°N, 102.25°E; and Site 4 = 72.75°N, 102.25°E) comparing only years 1934–2013.

## Electronic supplementary material

Supplementary Information

Supplementary Tables

## Data Availability

The Illumina sequences of the two datasets are submitted to the European Nucleotide Archive under project number PRJEB23161. Pollen data for the core from lake CH06 (11-CH-06D) is available at http://pangaea.de.
