# Supplementary material for: Temporal and spatial patterns of mitochondrial haplotype and species distributions in Siberian larches inferred from ancient environmental DNA and modeling
Source: Sci Rep. 2018 Nov 29;8:17436. doi: 10.1038/s41598-018-35550-w (PMC6265258; doi:10.1038/s41598-018-35550-w)
Supplement: Supplementary file 1 — Supplementary Information [file 41598_2018_35550_MOESM1_ESM.docx]

**Temporal and spatial patterns of mitochondrial haplotype and species distributions in Siberian larches inferred from ancient environmental DNA and modeling**

**Supplementary information**

Authors: Laura S. Epp^1^, Stefan Kruse^1^, Nadja J. Kath^1,2^, Kathleen R. Stoof-Leichsenring^1^, Ralph Tiedemann^2^, Luidmila A. Pestryakova^3^, Ulrike Herzschuh^1,2,4^

Postal addresses

^1^ Alfred Wegener Institute Helmholtz Centre for Polar and Marine Research, Periglacial Research Section, Telegrafenberg A43, 14473 Potsdam, Germany

^2^ Institute for Biochemistry and Biology, University of Potsdam, Karl-Liebknecht-Str 24-25, 14476 Potsdam, Germany

^3^ Department for Geography and Biology, North-Eastern Federal University of Yakutsk, Belinskogo 58, 67700 Yakutsk, Russia

^4^ Institute of Earth and Environmental Science, University of Potsdam, Karl-Liebknecht-Str 24-25, 14476 Potsdam, Germany

1 Details of the model

*1.1 Model modification overview*..................................................................................2

*1.2 Climate input pre-processing..................................................................................*2

*1.3 Introduction of a second species besides* L. gmelinii*..............................................*2

*1.4 Adapting the diameter growth function*...................................................................3

*1.5 Mortality and ageing...............................................................................................*4

*1.6 Seed dispersal*..........................................................................................................4

*Table S1.........................................................................................................................*5

2 Dating sediment core 11-CH-06D.....................................................................................6

3 Genetic analyses of the sediment cores

*3.1 Sampling for DNA*...................................................................................................6

*3.2 Laboratory work*......................................................................................................6

*3.3 Sequence analyses*...................................................................................................8

4 Detailed results of amplicon sequence data

*4.1 Plant metabarcoding*...............................................................................................9

*4.2 Mitochondrial nad4*.................................................................................................9 *Table S6*.......................................................................................................................11

5 Stratigraphic figures...........................................................................................................12

6 Principal component analyses...........................................................................................16

References...............................................................................................................................18

Tables S2 - S5 as Excel files

*Table S2 Ages of sediment core 11-CH-06D*

*Table S3 Terrestrial vascular plant metabarcoding data of both cores*

*Table S4 Non-authentic plant DNA sequences*

*Table S5 Mitochondrial nad4 amplicon sequences*

## 1 Details of the model

### 1.1 Model modification overview

The individual-based spatially-explicit model LAVESI model was run with the general set of parameters used by ^1^ with some modifications to allow for a second tree species, *Larix sibirica* alongside *Larix gmelinii*, to establish (Table S1).

### 1.2 Climate input pre-processing

Quasi-daily temperature data $T_{i}$ were estimated from monthly temperature series based on a sine function describing the temperatures based on the coldest and warmest month January (Jan) and July (Jul) which were used to calculate a weighted mean

$T_{i}=\left( a*\sin\left( b*\left( \frac{i}{\frac{365}{12}} \right)+c \right) \right)-d+T_{month}$, with a = 21.73946 (Jan) and 21.77314 (Jul), b = 0.45007 (Jan) and 0.44797 (Jul), c = 4.69717 (Jan) and 4.69882 (Jul), and d = 21.09023 (Jan) and 20.83202 (Jul).

Due to mismatches between the auxiliary variables ‘active air temperature’ (AAT), ‘vegetation length’ (NDD) and ‘degree days temperature’ (DDT), correction factors were derived by comparing the estimated variables and the climate series: AAT: 0.68650, NDD: 0.7511 and DDT: 0.71502. These were directly used and not further processed as in the original publication.

The January temperature and an auxiliary variable determining a growth restriction is now species-specifically implemented by the function $1-\left| 9*\frac{\bar{T_{Jan}}+45}{45} \right|$ for *L. gmelinii* and for *L. sibirica* by $1-\left| 6.6*\frac{\bar{T_{Jan}}+33}{33} \right|$.

### 1.3 Introduction of a second species besides *L. gmelinii*

To differentiate the two species *L. sibirica* and *L. gmelinii,* each of the seeds and trees carry an additional parameter called ‘species’, and the general presence of species in each run was defined by a parameter called ‘specpres’. The parameter ‘species’ is either set during initial tree establishment by deliberate insertion of the seed of a species, or, otherwise, is inherited by its parental tree. It determines a set of other parameters associated with each individual. Species-specific values of the parameters for seed dispersal, seed production, and mortality were compiled from literature sources (Table S1).

### 1.4 Adapting the diameter growth function

The values for the growth function were determined by selecting climate variables influencing tree growth by correlation analysis between tree ring width series and climate data within two regions of monospecific forests. For *L. sibirica*, these are the tree ring series ‘RUSS223’ at 67.5° N and 70° E from the National Climatic Data Center data bank ^2^ and the closest weather station Yamal (1934-2013; 67.30° N, 70.00° E). For *L. gmelinii,* data from the region in the vicinity of the settlement Khatanga was employed ^3^, and the closest weather station for comparison is located in Khatanga (1934-2013; 71.98° N and 102.47° E). The set of six climate variables that were initially included (temperatures: January, June, July, spring (Mar-May); precipitation: winter (Dec-Feb) sum, annual sum) were reduced by stepwise exclusion of climate variables to retain the most parsimonious model based on the Bayesan Information Criterion (BIC) via backward regression ^4^. This produced a model, which depends on July temperatures only.

The possible diameter growth under actual climate is thus estimated by a logistic growth form independently for the two species by $\hat{G}_{temp}=\left( \frac{a}{1+e^{b-T_{Jul}}} \right)+c$, with a = 0.163 (*L. gmelinii*) or 0.078 (*L. sibirica*), b = 12.319 (*L. gmelinii*) or 14.825 (*L. sibirica*) and c = 0.168 (*L. gmelinii*) or 0.108 (*L. sibirica*). Furthermore, each tree can grow by a certain amount in diameter which depends on the realised growth of the previous year described by $\hat{G}_{ind}(t+1) = e^{a+b*DIA(t)-c*{DIA(t)}^{2}}$, with a = -1.0111 or -1.9829, b = 0.0056 or 0.0303, DIA current diameter, and c = -0.0009 or -0.003 (Table S1).

The individual tree’s growth is lowered by competition with other individuals using the basal diameter value in the computations (parameter ‘densitytreetile’ set to 1) and not by a relative value between 0 and 1 as in the original version. Furthermore, to compute the competition a tree is experiencing, each value in the density grid is multiplied by the influence of the tree on this grid cell and not summed (parameter ‘densitytiletree’ set to 1).

This is further constrained by the active layer thickness (ALT). The ALT is estimated in metres for each year with the Stefan Formula, incorporating published simplifications ^5^. It is determined by soil properties $fe$ = 0.050 (Global Land Cover Characterization^6^) and the cumulative sum of daily temperatures exceeding the freezing points DDT: $ALT\left( year \right)=1.0-fe*\sqrt{DDT\left( year \right)}$. The diameter growth for each individual is based on ^7^. It describes a linear relationship allowing 100% diameter growth at 100 cm thawing depth and only 10% when reaching 10 cm, the minimum value for *L. gmelinii*. For *L. sibirica* it reaches 10% at 100 cm and follows the function for *L. gmelinii*.

### 1.5 Mortality and ageing

Influence of growth on mortality *mgrowth* is set to zero in the updated version as the actual growth of a tree depends on the density of surrounding trees and the weather which are already included in the mortality function. The mortality dependency on weather forcing and age (*myouth*) is now calculated specifically for each species (Table S1).

Longevity of seeds was set to two years for *L. gmelinii* ^8^ and to ten years for *L. sibirica* ^9^.

### 1.6 Seed dispersal

Each seed is distributed in a random direction with a ballistic maximal flight distance $E_{0}=V_{h}\frac{H_{t}}{V_{d}}$ estimated by species-specific size parameters following the approach of ^10^. The parameter $V_{h}$ is defined as the mean horizontal wind speed of 10 km/h and the release height $H_{t}$ is roughly estimated as 75% of the individual’s height of the releasing tree. $V_{d}$ is the descent rate for seeds and is estimated by a linear regression using species-specific data ^10^ of those taxa having wing-scales attached to the seeds similar to larches: $V_{d} =0.0032*\sqrt{w}+0.4807$ and is 0.86 or 0.93 m/s for *L. gmelinii* or *L. sibirica*, with the wing loading $w$ ^10^ calculated by dividing the average seed weight (in microdyne) of 3.5 or 10 mg ^11-13^ by the propagule area of 0.2 cm^2^ ^14^. This variable $E_{0}$ controls the standard deviation of the Gaussian term ${DL}_{gaussian}$ in the dispersal function ${DL}_{gaussian with fat-tail}\left( rn \right)$ of the model, which is named originally ‘*width*’ in Equation 5 of the original publication ^1^, and reads: ${DL}_{gaussian}\left( rn \right)=\sqrt{2*{E_{0}}^{2}*-1*log\left( rn \right)}$, with $rn$ – random number uniformly distributed between 0 and 1. Finally, actual dispersal distance $DL$ is normalised to the 99.9% quantile of maximum possible seed dispersal in the model (=4500 m) by $DL=\left( \frac{E_{0}}{4500 m} \right)*{DL}_{gaussian with fat-tail}$.

**Table S1: Parameters, which were modified for the updated version of the model LAVESI.** See detailed description for all parameters and parameter adjustments in Table 3 of ^1^.

| **Parameter** | | **Value and dimension** | **References** |
| --- | --- | --- | --- |
| *Growth* | |  |  |
| * | Growth function $\hat{G}_{ind}$for *L. gmelinii* and *L. sibirica* at basal and breast height  a  b  c | -1.98 and -1.01  0.030 and 0.0056  -0.003 and -0.0009 | Data-based estimates |
|  | Precipitation optimum | 250 mm | Estimated |
| *Seed production, dispersal and establishment* | |  |  |
|  | Dispersal mode: Gaussian with fat-tail with height-dependency | 5 | Functions follow ^10,15^ |
| * | Dispersal distance of *L. gmelinii* and *L. sibirica* seeds travelling in mean winds of 10 km/h | 60.1 and 45.0 m | Calculation follows ^10^ |
| * | Descent rate of *L. gmelinii* and *L. sibirica* | 0.86 and 0.93 m∙s^-1^ | Calculation follows ^10^ |
|  | Factor of seed productivity | 7 | Literature-based estimate cited in ^13,16^ |
|  | Seed maximum age for *L. gmelinii* and *L. sibirica* | 2 and 10 yrs | ^8,9^ |
|  | Probability of seed release from cones | 0.7 | Estimated |
|  | Factor of dispersal distance | 0.16 | Literature-based estimate ^13^ |
|  | Background germination rate | 0.0 | Estimated |
|  | Influence factor of weather on germination rate | 0.5 | Estimated |
| *Mortality* | |  |  |
|  | Background mortality rate | 0.0005 yr^-1^ | Data-based estimate |
|  | Tree youth influence factor on tree mortality | 0.25 | Estimated |
|  | Mean temperature of coldest month that *L. gmelinii* and *L. sibirica* can endure | -45 and -33 °C | ^17^ |
|  | Span of tree youth mortality | 0.25 | Data-based estimate |
|  | Influence factor for trees older than the age limit on tree mortality | 10 | Estimated |
|  | Current tree growth influence factor on tree mortality | 0.0 | Estimated |
|  | Weather influence factor on tree mortality | 0.0417 | Estimated |
|  | Density influence factor on tree mortality | 0.1 | Estimated |
|  | Drought influence factor on tree mortality | 0.3 | Estimated |
|  | Seed mortality rate on trees (in cones) and  at the ground | 0.8 yr^-1^ | Estimated ^13^ |

*Asterisks on the left indicate newly introduced parameters.*

## 2 Dating sediment core 11-CH-06D

The chronology of the sediment core from lake CH06 (11-CH-06D) is based on six ^14^C dates obtained from wood, moss, fruit, and bulk sediments at the *Poznan Radiocarbon Laboratory*. Age-depth modelling was performed using the Bacon package ^18^ in the R environment version 3.02 ^19^, basing the calibrated ages on IntCal13 ^20^. Recent sedimentation rates were inferred as 0.03 cm yr^-1^ for the last 180 years from ^210^Pb / ^137^Cs^-1^ dating of a parallel short core. Ages inferred from the age-depth model are compiled in Table S2.

## 3 Genetic analyses of the sediment cores

### 3.1 Sampling for DNA

Sampling for DNA was performed at 10°C in the climate chamber of the GFZ German Research Centre for Geoscience, a closed, windowless room in the cellar of the research institute, in which all benchtops were thoroughly cleaned with detergent, DNA-ExitusPlus™ (PanReac AppliChem, Germany), and water prior to sampling. DNA samples were taken from a core, half of which had been previously sampled for pollen. For sampling, the top of the cut surface of the core was removed with sterile scalpel blades, by lifting off in two cuts the topmost 0.5 cm of sediment around the sampled centimetre, as detailed in ^21^. Samples were taken with a sterile syringe, of which the top was cut with a cleaned knife. The knives were cleaned with 5% sodium hydroxide (VWR, Pennsylvania), rinsed with 96% technical ethanol (Carl Roth, Germany), and subjected to close distance UV irradiation for 10 minutes on each side in a CL1000 ultraviolett crosslinker (UVP, USA) ^22^.

### 3.2 Laboratory work

DNA extraction of most sediment core samples were carried out in a dedicated ancient DNA laboratory of the Alfred Wegener Institute in Potsdam (AWI), located in a building devoid of any other molecular genetic facilities. Extraction of three samples was performed in a dedicated laboratory for historical DNA at the University of Potsdam, working under a UV-hood used only for DNA isolation. Surface sediment samples were extracted in the pre-PCR laboratory of AWI, working under a UV-hood dedicated to the extraction of environmental samples. Extraction of total DNA was carried out using the PowerMax™ Soil DNA Isolation Kit (MOBIO), with the addition of 0.8 mg proteinase K per extraction and overnight incubation at 56 °C in the lysis step. Each extraction round, comprising between 3 and 10 samples, included one extraction blank containing only the chemicals. PCR setup of the sediment core samples was carried out in the was carried out in the ancient DNA laboratory of the AWI in Potsdam, while for the surface samples this was done under a separate UV-hood in the general pre-PCR laboratory. PCR reactions were performed in 25 µl volumes containing 1.25 U Platinum® *Taq* High Fidelity DNA Polymerase (Invitrogen), 1x PCR buffer, 2 mM MgSO_4_, 1 mM dNTPs, 0.2 μM of each primer, 0.8 mg/ml Bovine Serum Albumin (BSA) and 3 µl DNA extract. PCR reactions were conducted using 1) the primers trnL-g and trnL-h ^23^ targeting all vascular plants, and 2) primers specific to *Larix*, targeting a short amplicon of 61 base pairs without primers around the variable position 1433 of the mitochondrial *nad4/3-4* amplicon, where *Larix* individuals from the range of *L. sibirica* show an A and individuals from the range of *L. gmelinii* show a C ^24^. The new primers were nad4_1398_F: ACCATACCATATCAGGTGCCAG and nad4_1481_R: CAGCGCCACGATCTGA-TCTC, T_a_ 60°C. To sort sequences to samples after sequencing, the primers were modified as in Paus *et al.* (2014), carrying unique 8 bp tags on the 5' end that differed from each other in at least 5 bp, and were preceded by NNN to improve cluster generation on the sequencer (Coissac 2012; De Barba *et al.* 2014). PCRs were run with the following conditions: 2 min at 94°C, followed by 50–60 cycles of 94°C for 30 sec, T_a_ for 30 sec, 68°C for 30 sec, and final extension for 10 min at 72°C. For each sample and primer pair, amplification was attempted up to five times. Two positive products were pooled, purified using the MinElute PCR Purification Kit (Qiagen) and quantified using a Qubit 2.0 fluorometer (Invitrogen). Equimolar mixtures of PCR products of the differenct experiments were sequenced on 10% of an Illumina HiSeq lane (paired-reads, 2x125 bp) by an external sequencing service, Fasteris SA (Switzerland). The sequencing run of the trnL PCR products included products generated both from the cores and from surface sediments published in ^25^. The sequencing run of the nad4-products contained a subset of these samples, for which positive amplifications were obtained, and the run was performed including a second set of PCR products generated with another primer (not discussed here). Although no contamination was observed after visual inspection of the PCR products on an agarose gel, and all blanks, both from the extraction and the PCR, remained negative, we took a conservative approach and sequenced all PCR reactions from extraction blanks and PCR blanks.

### 3.3 Sequence analyses

The program *illuminapairedend* was used to assemble forward and reverse reads, followed by assigment of reads to samples by exact matches with their tags using *ngsfilter.* Reads shorter than 10 bp were excluded using *obigrep*, and using *obiuniq* identical reads were merged, keeping information on the number of original reads and occurence of these sequences in samples. Putative PCR and sequencing errors were identified and removed using *obiclean*, keeping only sequences that were identified as "head" or "singleton" in the dataset ^26^. Finally, sequences occurring less than 10 times in a sample were removed from the dataset to mitigate effects from tag-switching ^27^.

For the metabarcoding data generated with the primers trnL g and trnL h, taxonomic assignment and choice of taxa included in downstream analyses was performed as in ^25^, considering only sequences with a best identity of 1 to one of the reference databases used. To avoid potential contaminants, we excluded all sequences that were identical to sequences from food plants or cultivated plants, and we checked extraction blanks and PCR negative controls for sequences that were present in high numbers, and excluded these sequences. This was only the case for four sequence types from a PCR control and one sequence type from an extraction blank. A further extraction blank contained a low number of sequences of a total of 11 types, but these are more likely due to tag-switching ^27^ than to contamination, and we did not discard these taxa. Details of the sequencing results are compiled in the Supplementary information, and full lists of the vascular plant taxa and MOTUs, including the potential contaminants, are compiled in Tables S2 and S3.

The sequence data for the fragment of the mitochondrial *nad4* amplicon was analysed with the OBITools package as described above, except for filtering of fragment length, where we kept only sequences with lengths between 55 bp and 65 bp. The filtered sequences were then taxonomically assigned, and they were compared to the two *Larix* haplotype sequences known from tree samples ^24^. For taxonomic assignments, we first ran *ecoTag* using a reference library based on the embl standard sequences release 117 and created by running ecoPCR run with the primers nad4_1398_F and nad4_1481_R (five mismatches allowed). This creates a restricted sequence database for identification, and therefore, for a complete taxonomic overview of the sequencing output we additionally performed an online BLASTn search against GenBank, downloading the 10 best hits. These were parsed using the MEGAN6 Community Edition ^28^ to assign each read to the lowest common ancestor among the BLASTn hits. We then aligned all sequences assigned to *Larix* by ecoTag to published representative sequences of the two haplotypes (GenBank Acc. Nos. FJ572134 and FJ572140). We assigned all sequences that differed by no more than one nucleotide position to the reference sequences (equivalent to a cutoff value of 98% identity) to one of the two haplotypes and considered in the further interpretations of haplotype presence.

## 4 Details of the amplicon sequence data

### 4.1 Plant metabarcoding

Sequencing of the plant metabarcoding data of the two cores was performed on a run that included additional samples not discussed here. The complete sequencing run resulted in a total of 35,805,363 sequence reads after joining forward and reverse sequences with *illuminapairedend*. For the core samples alone, we retained 1797 unique sequence types and a total of 18,880,417 reads after filtering for length, merging unique sequences, removing potential PCR errors after *obiclean* analyses, and discarding sequences present less than 10 times. Within these, we identified 228 sequence types, corresponding to 14,187,136 reads, that had a best identity of 1 to either of the two references bases used (I: for Arctic and Boreal taxa and II: based on the complete embl standard sequence release 117 ^25^). A total of 151 sequence types (from 3,124,328 reads) were identified as terrestrial taxa, including Cyperaceae, and considered to be authentic. A full list of these is compiled in Table S3, while sequences that were not considered authentic and therefore discarded, are compiled in Table S4. The latter comprises 33 sequence types, with over 99% of reads (2,669,785) belonging to two sequence types that were nearly exclusively found in one of the NTCs, and were assigned to *Poa* and *Pisum* by the embl117 sequence database.

### 4.2 Mitochondrial nad4

The mitochondrial nad4 amplicon data were sequenced in a sequencing run together with another marker, not described here. The complete sequencing output after running *illuminapairedend* were 9,962,400 reads, of which 4,867,968 reads were assigned to the nad4 experiment. After filtering for length, merging unique sequences, removing potential PCR errors after *obiclean* analyses, and discarding sequences present less than 10 times, we retained 216 unique sequence types and a total of 3,776,112 reads (full overview compiled in Table S5). Of these, 78 sequences types, from 2,775,722 reads, were assigned to *Larix* by *ecotag,* with 30 sequence types, from 2,763,203 reads, showing a best identity of at least 98% to the existing *Larix* reference sequences. These sequences were assigned to each of the two variants (Table S6). A further 48 sequence types were assigned to *Larix* with a lower best identity value, but these comprised only 12,519 additional reads. All our extraction blanks (for core and surface samples) contained no sequences, and only very few numbers of sequences that were not retrieved in any of the samples were recorded in one of the NTCs, indicating that no substantial contamination was introduced in the laboratory.

The three topmost samples from core CH12 contained sequences assigned to *Pinus*, and the sample taken at 14.5 cm depth did not yield any *Larix* sequences. Similarly, one of the three replicates from the sample at depth 28.5 cm yielded no *Larix* sequences, and the two remaining replicates differ with respect to the outcome regarding the dominance of *Larix* haplotypes. One replicate shows a mixed signal, while the other is dominated by the "*L*. *gmelinii*"-variant. Both the retrieval of *Pinus* DNA from these samples, as well as the uncertain result of haplotype dominance is probably due to the extremely low amount of *Larix* DNA. A similar occurrence of *Pinus* DNA to the exclusion of *Larix* was observed in two of the surface sediment samples, and seven further surface samples recorded only non-Pinaceae sequences. As stated above, we do not think that these were the result of laboratory contamination, but rather contained within the samples. Such sequences were not apparent in the core samples, and although we sampled with care, we cannot rule out contamination during field sampling of the surface samples. We nonetheless consider the overall pattern of spatial and temporal distribution of the haplotypes to be reliable.

**Table S6: Summary of the nad4-variant assignment for each of the samples.**

Due to the defining nucleotide difference in the amplicon, the two *Larix* variants are here named Larix A ("*L*. *sibirica*" variant) and Larix C ("*L*. *gmelinii*" variant)

| **Lake** | **Depth (cm)** | **Replicate** | **Lat** | **Long** | **Sum *Larix* A** | **Sum *Larix* C** | **Dominating** |
| --- | --- | --- | --- | --- | --- | --- | --- |
| 11-CH-12 | 0.5 | / | 72.40 | 102.29 | 16 | 73830 | C |
| 11-CH-12 | 14.5 | / | 72.40 | 102.29 | 0 | 0 | *Pinus* |
| 11-CH-12 | 28.5 | 1 | 72.40 | 102.29 | 0 | 0 | *Pinus* |
| 11-CH-12 | 28.5 | 2 | 72.40 | 102.29 | 15 | 70066 | C |
| 11-CH-12 | 28.5 | 3 | 72.40 | 102.29 | 38154 | 36552 | mix |
| 11-CH-12 | 42.5 | / | 72.40 | 102.29 | 62790 | 47868 | mix |
| 11-CH-12 | 55.5 | / | 72.40 | 102.29 | 18854 | 11596 | mix |
| 11-CH-12 | 69.5 | / | 72.40 | 102.29 | 51330 | 70684 | mix |
| 11-CH-12 | 83.5 | / | 72.40 | 102.29 | 39224 | 32490 | mix |
| 11-CH-12 | 95.5 | / | 72.40 | 102.29 | 60104 | 73057 | mix |
| 11-CH-12 | 111.5 | / | 72.40 | 102.29 | 29 | 220458 | C |
| 11-CH-12 | 125.5 | 1 | 72.40 | 102.29 | 43565 | 215953 | mix |
| 11-CH-12 | 125.5 | 2 | 72.40 | 102.29 | 18311 | 10743 | mix |
| 11-CH-12 | 125.5 | 3 | 72.40 | 102.29 | 141128 | 84074 | mix |
| 11-CH-06 | 1 | / | 70.67 | 97.72 | 11 | 33090 | C |
| 11-CH-06 | 16 | / | 70.67 | 97.72 | 89 | 369194 | C |
| 11-CH-06 | 29 | 1 | 70.67 | 97.72 | 62043 | 9585 | mix |
| 11-CH-06 | 29 | 2 | 70.67 | 97.72 | 65922 | 4684 | mix |
| 11-CH-06 | 29 | 3 | 70.67 | 97.72 | 53201 | 34389 | mix |
| 11-CH-06 | 41 | / | 70.67 | 97.72 | 11 | 45041 | C |
| 11-CH-06 | 59 | / | 70.67 | 97.72 | 23 | 97628 | C |
| 11-CH-06 | 73 | / | 70.67 | 97.72 | 26535 | 5514 | mix |
| 11-CH-06 | 93 | / | 70.67 | 97.72 | 168720 | 26 | A |
| 11-CH-06 | 108 | 1 | 70.67 | 97.72 | 44123 | 15508 | mix |
| 11-CH-06 | 108 | 2 | 70.67 | 97.72 | 14963 | 22177 | mix |
| 11-CH-06 | 108 | 3 | 70.67 | 97.72 | 10483 | 34588 | mix |
| 11-CH-06 | 124 | / | 70.67 | 97.72 | 6240 | 0 | A |
| 11-CH-06 | 155.5 | / | 70.67 | 97.72 | 56971 | 0 | A |
| 13-TY-01 | 0.5 | / | 72.67 | 105.88 | 7907 | 0 | A |
| 13-TY-02 | 0.5 | 1 | 72.55 | 105.72 | 6840 | 0 | A |
| 13-TY-05 | 0.5 | / | 72.55 | 105.75 | 7929 | 0 | A |
| 13-TY-06 | 0.5 | 3 | 72.54 | 105.76 | 12 | 73433 | C |
| 13-TY-17 | 0.5 | / | 71.40 | 102.28 | 0 | 8513 | C |
| 13-TY-22 | 0.5 | / | 71.10 | 100.85 | 0 | 17285 | C |
| 13-TY-24 | 0.5 | / | 71.10 | 100.80 | 3634 | 23783 | mix |
| 13-TY-30 | 0.5 | / | 72.14 | 102.09 | 1889 | 10328 | mix |
| 13-TY-04 | 0.5 | / | 72.55 | 105.74 | 0 | 0 | *Pinus* |
| 13-TY-21 | 0.5 | / | 71.11 | 100.82 | 0 | 0 | *Pinus* |
| 13-TY-02 | 0.5 | 2 | 72.55 | 105.72 | 0 | 0 | Non Pinacea |
| 13-TY-02 | 0.5 | 3 | 72.55 | 105.72 | 0 | 0 | Non Pinacea |
| 13-TY-12 | 0.5 | / | 72.41 | 105.45 | 0 | 0 | Non Pinacea |
| 13-TY-06 | 0.5 | 1 | 72.54 | 105.76 | 0 | 0 | Non Pinacea |
| 13-TY-06 | 0.5 | 2 | 72.54 | 105.76 | 0 | 0 | Non Pinacea |
| 13-TY-08 | 0.5 | / | 72.49 | 105.65 | 0 | 0 | Non Pinacea |
| 13-TY-10 | 0.5 | / | 72.41 | 105.44 | 0 | 0 | Non Pinacea |

## 5 Stratigraphic figures

Figure S2: Stratigraphic plot of the DNA metabarcoding results of core CH12.

Figure S3: Stratigraphic plot of the pollen data for the samples used in this study of core CH12 data from ^29^.

Figure S4: Stratigraphic plot of the DNA metabarcoding results of core CH06.

Figure S5: Stratigraphic plot of the pollen data of core CH06.

## 6 Principal component analyses

Figure S6: PCA biplot of DNA metabarcoding data for core CH12. Only taxa that were present at least twice with a percentage of at least 0.5% were included.

Figure S7: PCA biplot of the pollen data for the considered samples from core CH12, data from ^29^.

Figure S8: PCA biplot of DNA metabarcoding data for core CH06. Only taxa that were present at least twice with a percentage of at least 0.5% were included.

Figure S9: PCA biplot of pollen data from core CH06.

## References

1 Kruse, S., Wieczorek, M., Jeltsch, F. & Herzschuh, U. Treeline dynamics in Siberia under changing climates as inferred from an individual-based model for Larix. *Ecological Modelling* **338**, 101-121, doi:10.1016/j.ecolmodel.2016.08.003 (2016).

2 Briffa, K. R. *et al.* Reassessing the evidence for tree-growth and inferred temperature change during the Common Era in Yamalia, northwest Siberia. *Quaternary Sci Rev* **72**, 83-107, doi:10.1016/j.quascirev.2013.04.008 (2013).

3 Wieczorek, M. *et al.* Dissimilar responses of larch stands in northern Siberia to increasing temperatures – a field and simulation based study. . *Ecology*, doi:10.1002/ecy.1887 (2017).

4 Boogaart, K. G. V. d. & Tolosana-Delgado, R. *Analyzing compositional data with R*. (Springer, 2013).

5 Hinkel, K. M. & Nicholas, J. R. J. Active layer thaw rate at a boreal forest site in Central Alaska, USA. *Arctic Alpine Res* **27**, 72-80, doi:10.2307/1552069 (1995).

6 Zhang, T. Spatial and temporal variability in active layer thickness over the russian Arctic drainage basin. *J Geophys Res-Atmos* **110**, (D16) (2005).

7 Nakai, Y. *et al.* Eddy covariance CO2 flux above a Gmelin larch forest on continuous permafrost in Central Siberia during a growing season. *Theor Appl Climatol* **93**, 133-147, doi:10.1007/s00704-007-0337-x (2008).

8 Xu, H. & Ban, Y. The distribution of seeds in the soil and the sustainability of the seed band of Larix gmelini in Northern Daxinganling Mountains. *Acta Phytoecological Sinica* **20**, 28-34 (1996).

9 Savin, E. & Dugarzhav, C. The number of larch seeds in the soil in the larch forests of Khangai. *Lesovedenie* **2**, 78–82 (1980).

10 Matlack, G. R. Diaspore Size, Shape, and Fall Behavior in Wind-Dispersed Plant-Species. *Am J Bot* **74**, 1150-1160, doi:10.2307/2444151 (1987).

11 Heit, C. E. & Eliason, E. J. Coniferous tree seed testing and factors affecting germination and seed quality. *Technical Bulletin. New York State Agricultural Experiment Station*, 45 pp. (1940).

12 Lukkarinen, A. J., Ruotsalainen, S., Nikkanen, T. & Peltola, H. The Growth Rhythm and Height Growth of Seedlings of Siberian (Larix sibirica Ledeb.) and Dahurian (Larix gmelinii Rupr.) Larch Provenances in Greenhouse Conditions. *Silva Fenn* **43**, 5-20, doi:10.14214/Sf.215 (2009).

13 Abaimov, A. P. Geographical Distribution and Genetics of Siberian Larch Species. *Ecol Stud-Anal Synth* **209**, 41-58, doi:10.1007/978-1-4020-9693-8_3 (2010).

14 Fu, L., Li, N. & Mill, R. in *Flora of China* (eds Z Wu & PH Raven) (Science Press 1999).

15 Nathan, R. & Muller-Landau, H. C. Spatial patterns of seed dispersal, their determinants and consequences for recruitment. *Trends Ecol Evol* **15**, 278-285, doi:10.1016/S0169-5347(00)01874-7 (2000).

16 Kruklis, M. & Milyutin, L. *Larix czekanovskii*. (Nauka, 1977).

17 Nikolov, N. & Helmisaari, H. in *A Systems Analysis of the Global Boreal Forest* (eds HH Shugart, R Leeman, & GB Bonan) (Cambridge University Press, 1992).

18 Blaauw, M. & Christen, J. A. Flexible paleoclimate age–depth models using an autoregressive process. *Bayesian Analysis* **6**, 457-474 (2011).

19 R: A Language and Environment for Statistical Computing. (Vienna, Austria, 2016).

20 Reimer, P. J. *et al.* *IntCal13 and Marine13 Radiocarbon Age Calibration Curves 0–50,000 Years cal BP*. (2013).

21 Epp, L. S., Zimmermann, H. H. & Stoof-Leichsenring, K. R. in *Ancient DNA: Methods and Protocols* (eds B. Shapiro *et al.*) (Springer Science+Business Media, in press).

22 Champlot, S. *et al.* An Efficient Multistrategy DNA Decontamination Procedure of PCR Reagents for Hypersensitive PCR Applications. *Plos One* **5**, doi:10.1371/journal.pone.0013042 (2010).

23 Taberlet, P. *et al.* Power and limitations of the chloroplast trnL (UAA) intron for plant DNA barcoding. *Nucleic Acids Res* **35**, e14, doi:10.1093/Nar/Gkl938 (2007).

24 Semerikov, V. L., Semerikova, S. A., Polezhaeva, M. A., Kosintsev, P. A. & Lascoux, M. Southern montane populations did not contribute to the recolonization of West Siberian Plain by Siberian larch (Larix sibirica): a range-wide analysis of cytoplasmic markers. *Mol Ecol* **22**, 4958-4971, doi:10.1111/mec.12433 (2013).

25 Niemeyer, B., Epp, L. S., Stoof-Leichsenring, K. R., Pestryakova, L. A. & Herzschuh, U. Recording vegetation composition at the Siberian boreal treeline: A comparison between sedimentary DNA and pollen. *Mol Ecol Resour*, doi:10.1111/1755-0998.12689 (2017).

26 Boyer, F. *et al.* OBITOOLS: a UNIX-inspired software package for DNA metabarcoding. *Mol Ecol Resour* **16**, 176-182, doi:10.1111/1755-0998.12428 (2016).

27 Schnell, I. B., Bohmann, K. & Gilbert, M. T. P. Tag jumps illuminated - reducing sequence-to-sample misidentifications in metabarcoding studies. *Mol Ecol Resour* **15**, 1289-1303, doi:10.1111/1755-0998.12402 (2015).

28 Huson, D. H. *et al.* MEGAN Community Edition - Interactive Exploration and Analysis of Large-Scale Microbiome Sequencing Data. *Plos Comput Biol* **12**, ARTN e1004957, doi:10.1371/journal.pcbi.1004957 (2016).

29 Klemm, J., Herzschuh, U. & Pestryakova, L. A. Vegetation, climate and lake changes over the last 7000 years at the boreal treeline in north-central Siberia. *Quaternary Sci Rev* **147**, 422-434, doi:10.1016/j.quascirev.2015.08.015 (2016).
